# Application of ERAS concept in clinical nursing of patients with advanced cancer pain of gynecological malignant tumors

**DOI:** 10.3389/fonc.2023.1173333

**Published:** 2023-09-25

**Authors:** Haijing Liao, Yuanxiu Lei

**Affiliations:** ^1^ Gynaecology Second area, First People’s Hospital of Chenzhou, Chenzhou, Hunan, China; ^2^ Gynaecology One area, First People’s Hospital of Chenzhou, Chenzhou, Hunan, China

**Keywords:** cancer pain, ERAS concept, malignant tumor, clinical nursing, clinical

## Abstract

Gynecological malignant tumors refer to malignant tumors of organs and tissues centered on the uterus, ovaries, and fallopian tubes. Among gynecological tumors, endometrial cancer is the most malignant, accounting for more than 80% of malignant tumors in the female reproductive tract. Common symptoms are vaginal bleeding and pain. This article aims to explore the application and analysis of the concept of ERAS (Enhanced Recovery After Surgery) in the clinical care of patients with advanced cancer pain from gynecological malignancies. ERAS aims to reduce complications, shorten hospitalization time, reduce medical costs, and enable patients to recover quickly by adopting a series of perioperative management measures for patients. This article analyzes the pain caused by late-stage cancer, proposes an ERAS multimodal analgesia method, and uses image fusion technology to detect cancer patients. This article finally conducts an experimental exploration of the clinical nursing of the ERAS concept in the treatment of advanced cancer pain in gynecological malignancies. The results of this study showed that in terms of pain impact score, before treatment, the score of group M was 39.07 and the score of group N was 38.92, and the difference was not statistically significant. The score after ERAS concept treatment was 58.14, and the score after traditional treatment was 43.79, with a significant difference. Research shows that the pain impact score after treatment is significantly better than before treatment, and the improvement effect of ERAS concept treatment is more obvious.

## 1 Introduction

Pain is the main symptom of patients with advanced cancer. In the process of disease diagnosis, clinicians usually take the “pain” of patients as an important diagnostic indicator to reduce the pain degree of patients through treatment. Recent studies have shown that chronic pain can be divided into three types: non-specific, physical pain and mental disorders. Advanced cancer pain is a series of chronic and severe pain caused by non-specific malignant tumors. Therefore, effective relief of non-specific cancer pain is of great significance for prolonging the survival of patients with advanced cancer. Cancer pain is different from some chronic pain. It rarely has an intermittent period. Once the cancer progresses, the pain would become more and more severe, and the degree of tissue damage would become more and more serious. Analgesia for advanced cancer has always been the focus of clinicians and nursing staff. With the transformation and progress of medical technology and medical model, ERAS concept has been widely applied and promoted worldwide. The ERAS concept emphasizes the use of evidence-based medical evidence to guide clinical treatment and nursing practice, which can provide personalized pain care programs for cancer patients. The purpose of this article was to explore the application and analysis of ERAS concept in the clinical nursing of patients with advanced cancer pain of gynecological malignant tumors, with a view to making certain contributions to the relief of cancer pain.

Based on the existing research results, scholars have carried out relevant research on cancer pain. Pain is a common symptom of cancer patients. Appropriate pain evaluation and treatment is the key to improve the quality of life and health effect of patients. Scarborough Bethann M. reviewed pain control, pain management obstacles, evaluation and management strategies of tumor-related pain, pain management of patients at risk of drug abuse, and problems that should be paid attention to in pain management of patients (1). Pain was a common and painful symptom of childhood cancer, as reported by children and their parents. Nowadays, more and more cancer children were treated as outpatients, but little is known about how parents treat the pain caused by cancer. Tutelman Perri R.’s research aimed to explore the pain symptoms of children, and explore the drug, physiological and psychological treatment methods adopted by parents in the treatment of children’s tumor pain (2).

There are more and more functions of acupuncture and acupoint massage in tumor pain, but the results of these studies are different. He Yihan’s purpose was to determine the relevant evidence of acupuncture and massage for tumor pain relief by evaluating the existing randomized clinical trials (3). There was increasing interest in the use of cannabinoids in the treatment of chronic non-cancer pain, as they may reduce the demand for opioid doses. Campbell Gabrielle aimed to investigate the use of cannabis in patients with chronic non-cancer pain who take opioids (4). However, these scholars have not discussed the treatment of cancer pain. Through research, it is found that ERAS concept is better for the relief of cancer pain. In this regard, relevant literature on ERAS concept was consulted.

Some scholars also have some research on ERAS concept. The concept of ERAS is applied to different surgical fields. Among the patients undergoing radical cystectomy, there is still a lack of prospective data. A prospective randomized study showed that ERAS has more advantages over traditional treatment for these patients. Ziegelmueller Brigitte Katharina aimed to evaluate the long-term follow-up effect of ERAS after radical cystectomy (5). At present, the method of using ERAS in thoracic surgery was still novel, and the reference data were also few. The purpose of Rogers Luke J was to explore the impact of strengthening rehabilitation approaches for tumor patients after surgery on the incidence rate and hospitalization period of lung cancer patients (6). Although it has clinical benefits, the intensive treatment of ERAS has not been widely used in tumor surgery. Jeong Oh studied the application of ERAS in surgery for gastric cancer patients in South Korea (7). However, these scholars did not discuss the application and analysis of ERAS concept in the clinical nursing of patients with advanced cancer pain of gynecological malignant tumors, but only discussed it from a superficial level.

In order to alleviate cancer pain caused by advanced malignant tumor and cancer pain caused by various reasons, this paper screened out gynecological malignant tumor patients through medical image fusion technology, and analyzed the causes of advanced cancer pain. In this paper, the ERAS multimodal analgesia method was proposed, and based on this, the clinical nursing simulation experiment of gynecological malignant tumor and advanced cancer pain was designed. It can be seen from the experimental results that ERAS concept had a good effect in the nursing of patients with advanced cancer pain of gynecological malignant tumors.

## 2 Methods of ERAS concept in clinical nursing of advanced cancer pain

### 2.1 Advanced cancer pain

Pain is a kind of sensation, and its occurrence and development are mainly regulated by nerves and body fluids. These neurotransmitters or factors participate in central sensitization through corresponding signal transduction pathways. The physiological classification of pain is based on the reasoning of the mechanism of pain, which is divided into injury sensation type and non-injury sensation type. Nociceptive pain can be divided into physical pain and visceral pain. There are two types of non-invasive sensory pain: neuropathic pain and psychological pain. When cancer causes cancerous pain, its mechanism of occurrence and development is as follows: when the body is in a state of stress, factors such as the body’s stress response, sympathetic nerve stimulation, and increased activity of pain receptors would cause corresponding changes in neurotransmitters and inflammatory factors (8, 9). Cancer pain is the biggest pain factor for patients with advanced cancer. It is caused by the transmission of information from the pain site to the nerve center. In addition, with the growth and metastasis of the tumor, the nerve endings at the tissue injury site are stimulated to produce excitatory neurotransmitters that cause pain. Therefore, cancerous pain is the result of multiple factors (10, 11).

**Figure 1:**
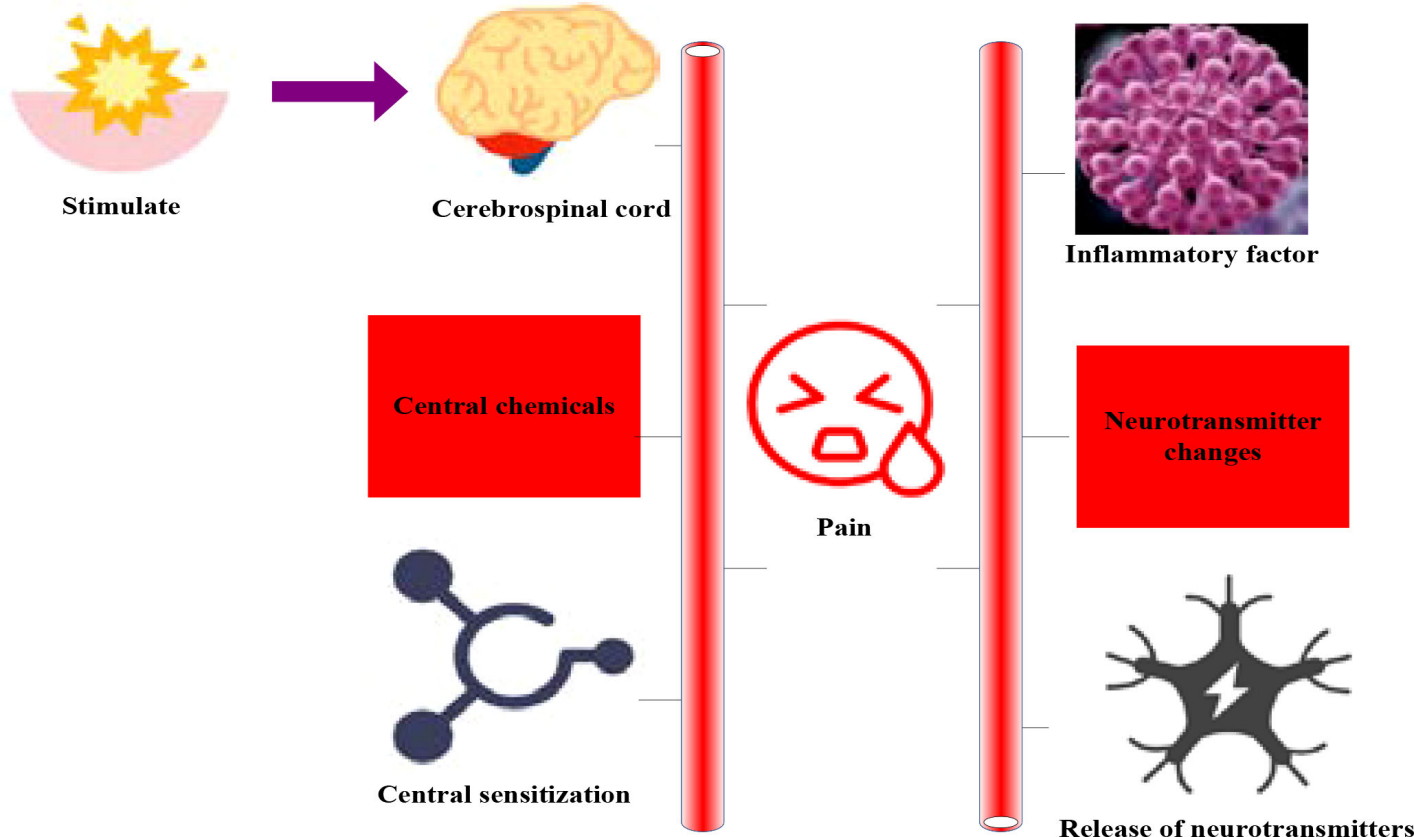
Basic mechanism of pain.

The basic mechanism of pain is as follows: the sensory nerve roots in the spinal cord and brain are stimulated to produce excitation or inhibition. Neurotransmitters such as dopamine and norepinephrine in the limbic system of the brain are released in varying degrees in the spinal cord and brain. The spinal cord and center would be sensitized, and the neurotransmitter changes (endogenous and exogenous). Central chemical substances act on peripheral organs or systems, inflammatory factors act on peripheral organs or systems, and ion channels or membrane protein abnormalities occur in pain cell membranes. **Figure 1** shows the basic mechanism of pain. Cancer pain is a physiological phenomenon, but it can also lead to psychological problems, such as anxiety, depression, fear, despair and other psychological abnormalities (12, 13). In addition, when the tumor spreads and metastases, it would also cause serious pain symptoms.

Pain is a subjective feeling of cancer patients. Therefore, patients would have problems in understanding, emotion and behavior of pain. When these problems affect the normal quality of life, they would affect their ability to live and work. The psychological burden of cancer patients mainly includes two aspects. On the one hand, it is anxiety, fear and other emotions caused by various physiological reactions that may occur during the treatment of the disease. On the other hand, it is the physical function damage caused by the physiological function disorder caused by pain. Because of the strong impact of cancer on psychology, the patients’ negative emotions such as depression and despair are generated. During this process, patients may suffer from insomnia, irritability, depression and other psychological abnormalities.

### 2.2 ERAS multimodal analgesia

The application of multimodal analgesia in postoperative pain management is an important part of ERAS. With the development of ERAS concept, multimodal analgesia technology has gradually been widely used. Multi-modal analgesia refers to the combination of multiple painkillers or different painkillers. It can reduce the amount of single analgesic, reduce the incidence of side effects, and improve the efficacy of surgical analgesia. Multi-mode analgesia includes preemptive analgesia, preventive analgesia, anesthesia and analgesia technology, postoperative analgesia, etc. The preemptive analgesia is to intervene before the nerve center has received the pain information caused by surgical trauma. It includes physical therapy or drug therapy to prevent hyperalgesia in the nerve center. Central hyperalgesia is characterized by hypersensitivity of pain receptors to stimuli. Preventive analgesia can significantly relieve postoperative pain and effectively prevent long-term postoperative pain. Anesthesia analgesia is a method of analgesia, which uses anesthetics to block the pain signals on the nerves. It includes local anesthesia, nerve block, fascial space anesthesia, intraspinal analgesia, etc. The purpose of multimodal analgesia is to effectively control the pain symptoms, reduce the stress response of patients after surgery, and accelerate the recovery of patients. The core principle of ERAS is to reduce the stress response of surgery and reduce the risk of complications through multimodal methods.

For cancer patients undergoing chemotherapy, the current mode of analgesia is relatively simple, and opioid analgesics such as morphine are usually used for severe pain. Effective pain management is an important part of ERAS concept, and multimodal analgesia can well control postoperative pain. Multi-modal analgesia can reduce the use of opiates and reduce the adverse reactions related to opiates. ERAS concept is a new perioperative treatment process. It is a new surgical method with the best perioperative treatment. It adopts a new set of technologies to reduce pain, cure patients and recover quickly.

ERAS concept is a combination of surgical technology and minimally invasive treatment concept, aiming to reduce the pain of patients, alleviate the physical and psychological pain of patients, and improve the quality of life. Through the application of ERAS concept in the nursing of advanced cancer pain, the analgesic effect is significant. The intervention measures and nursing process implemented by ERAS concept for patients with advanced cancer pain can be implemented, and the traditional surgical technology and minimally invasive technology can be combined to make painkillers and non-pharmaceutical analgesia methods be applied in the treatment of patients with advanced cancer pain. ERAS nursing mode plays an important role in the clinical practice of advanced cancer pain in patients with gynecological malignancies. It can effectively alleviate the adverse symptoms of patients at the physical and psychological levels, and can alleviate or eliminate -the side effects caused by chemotherapy drugs. It can also improve the quality of life and prolong the survival time of patients. ERAS nursing measures are mainly divided into analgesic drugs and non-drug treatment. Analgesics, non-analgesic sedatives, opioid analgesics and physical therapy can be used to relieve the pain of advanced cancer. Psychological counseling and psychotherapy: It can relieve the pain caused by advanced cancer through health education and psychological counseling.

### 2.3 Medical image fusion algorithm

In recent years, with the continuous development of computer technology, people pay more and more attention to the diagnosis of cancer, and the research on cancer is also deepening, especially some research related to human health. Cancer cell is a kind of mutant cell, which is the cause of tumor. Unlike ordinary cells, cancer cells have unlimited proliferation, transformation and transplantability. They can proliferate indefinitely and destroy normal cells. Cancer has a certain degree of heredity, and cancer cells have the characteristics of proliferation and spread, so cancer detection and recognition can provide more accurate information for early diagnosis of disease.

**Figure 2:**
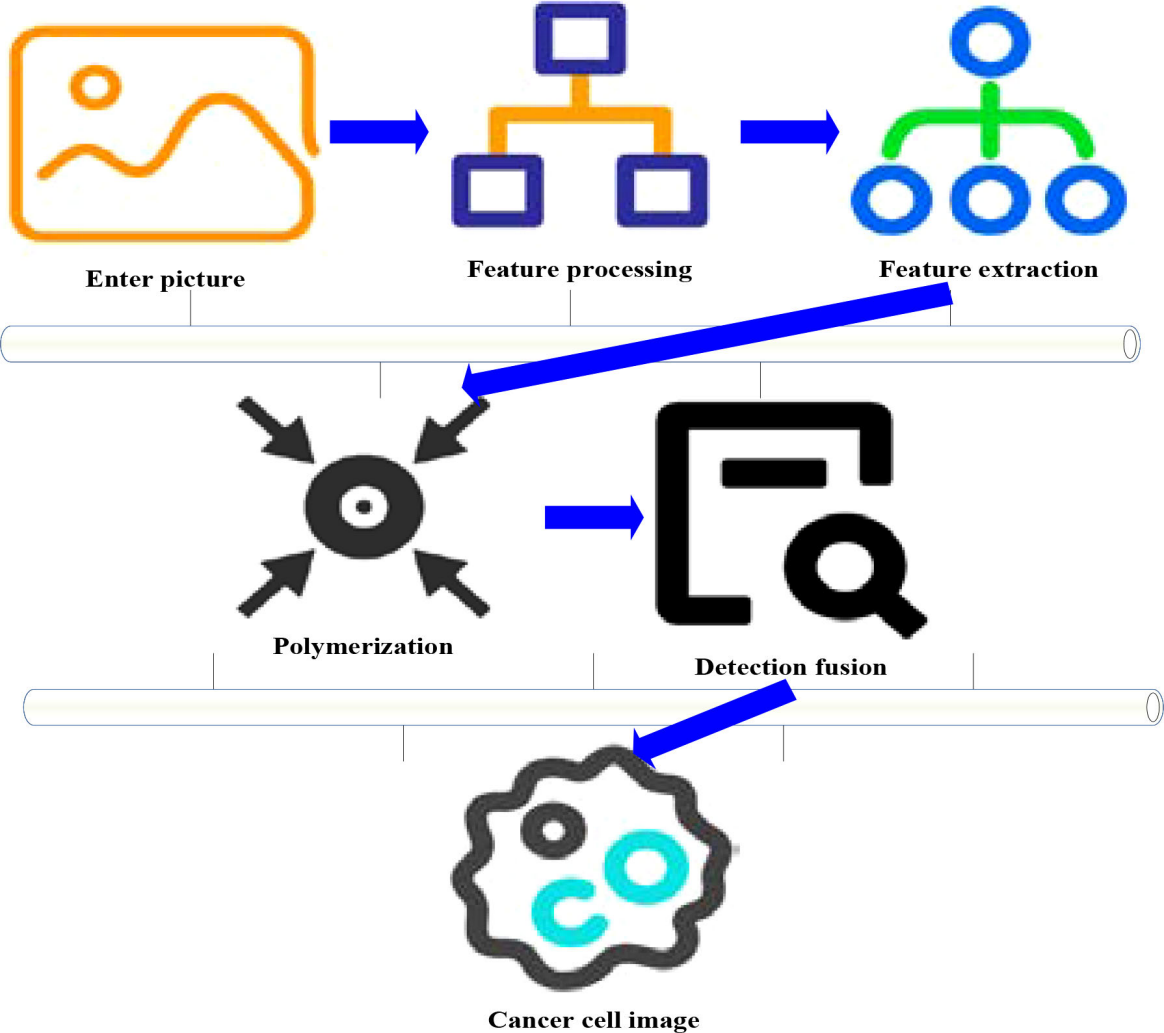
Application of medical image fusion in cancer.

The process of normal cells developing into tumors is called tumorigenesis. Tumor genesis is a gradual process, involving multi-level reaction and accumulation of mutations. The development of tumor would affect its surrounding normal tissues, and the changes of surrounding tissues are closely related to the metastasis and spread of tumor. Through multimodal medical images, the tissue information around the tumor can be obtained and transmitted to other organs, which is helpful for early detection and diagnosis of cancer. The application of fusion technology in malignant tumors mainly includes single-parameter fusion on magnetic resonance imaging (MRI) and fusion of MRI images using multi-layer convolution neural network. It also includes the layering of tumor, metastasis or normal tissue on electronic computed tomography (CT). The fusion can improve the sensitivity of the tumor edge and its surrounding blood vessels to a certain extent, thus improving the diagnostic accuracy. The fused image can better reflect the difference between malignant tumor cells and surrounding normal tissues in space, which provides better recognition for tumor sites. On the other hand, it can determine the scope of tumor more accurately and objectively, thus providing more accurate basis for tumor resection and follow-up treatment. **Figure 2** shows the application of medical image fusion in cancer. Medical image fusion is to combine the advantages of anatomical structure imaging and functional imaging to provide more and more accurate information for clinical use.

In some cases, the object of medical image fusion service is people. At this time, the purpose of fusion processing may be to improve the image quality to improve human visual effects. Or, this can increase the amount of information in the fused image or the accuracy and reliability of the information. This can provide richer, more accurate and more reliable image information for people’s decision-making. In other cases, the object of image fusion service is the machine, and the purpose of fusion processing may be to enable the machine or computer to automatically detect, recognize or track the target.

Because the target of image fusion is human, the evaluation of image is affected by human visual characteristics, psychological state, knowledge background, value orientation and other factors. Therefore, how to evaluate the effectiveness of image fusion, especially objectively and quantitatively, has very important practical significance.

At present, there is no unified and objective fusion image evaluation method. Here are several image fusion evaluation methods: cross entropy, interactive information, WFQI (Weighted Fusion Quality Index) to make an objective evaluation of the fusion image. The details are as follows:

(1) Cross entropy: it is also called correlation entropy, which can be used to measure the difference between two pictures. If the standard reference image is T and the fused image is G, the correlation entropy between the reference image T and the fused image G is:

(1) $$\text{CETG}=\sum {\;}_{\text{o}=0}^{\text{A}-1}{\text{Q}}_{\text{To}}\text{log}\frac{{\text{Q}}_{\text{To}}}{{\text{Q}}_{\text{Go}}}$$

A is the gray scale of the entire image, and Q_To_ is the ratio of the number of pixels with a gray value of To to the total number of pixels in the image. The lower the interaction entropy of the image, the better the fusion effect of the fused image and the reference image.

(2) MI (Mutual information): The mutual information MI(S, N, G) among images S, N, G is defined as follows:

(2) $$\text{MI(S},\text{N},\text{G)}=\sum {\;}_{\text{o}=0}^{\text{A}-1}\sum {\;}_{\text{k}=0}^{\text{A}-1}\sum {\;}_{\text{l}=0}^{\text{A}-1}{\text{Q}}_{\text{sng}}(\text{o},\text{k},\text{l})\text{log}\frac{{\text{Q}}_{\text{sng}}(\text{o},\text{k},\text{l})}{{\text{Q}}_{\text{sn}}(\text{o},\text{k}){\text{Q}}_{\text{g}}(\text{l})}$$

As defined by cross entropy, A is the total gray level of the image. The greater the amount of mutual information MI(S, N, G), the more information the fused image can obtain, so as to achieve better fusion effect.

Criteria for evaluation of fusion effect: when the image entropy, average gradient and standard deviation obtained by a certain fusion method are large, the cross entropy is small, and the mutual information is large, the fusion effect is good.

(3) WFQI

In WFQI, for the source image s, n, the importance of the fused image s in window e is expressed by d(s|e), which usually depends on the entropy, contrast and variance in window e. Local significant weight γ(e):


(3) γ(e)=d(s|e)/d(s|e)+d(n|e)

The global significance V(e) is:


(4) V(e)=max(d(s|e),d(n|e))

Then the global significance weight v(e) is:


(5) $$\text{v}(\text{e})=\text{V}(\text{e})/\sum_{{\text{e}}^{`}\in \text{E}}\text{V}({\text{e}}^{`})$$

WFQI emphasizes two aspects: the significance and weighting coefficient of each window e in the fused image. Important areas have better visual effects and richer information. Therefore, different windows cannot be treated equally. It should be the regions with higher significance that have larger weights, and vice versa.

With the increase of image quality index, the more information the image contains, the greater the importance of the image, and the better the visual effect.

### 2.4 Application of medical image fusion algorithm in radiotherapy

At present, the main applications of multimodal image fusion algorithms include two aspects. The first is to obtain a group of joint information with radiation dose and side effects through multimodal image fusion of image acquisition and information extraction during radiotherapy. In addition, the clinical prediction data obtained from the fusion results of different modes can be used for machine learning training to identify new targets or diseases. The fusion algorithm can be used to build a deep convolution neural network model for multi-source medical images to improve the segmentation, classification and recognition capabilities. This is of great significance for better realization of radiotherapy plan. The fused multimodal image information can be used for radiotherapy plan design, which can help to obtain more accurate treatment dose to achieve the optimal treatment effect. In the field of accurate tumor radiotherapy, accurate registration of multiple tumor parameters and location information and multiple image sequence information can be achieved through multiple image fusion methods to effectively control the tumor area and improve the radiation dose.

## 3 Clinical nursing experiment results of gynecological malignant tumor with advanced cancer pain

### 3.1 Test objects and methods

In recent years, with the promotion and deepening of ERAS concept, more and more research evidence shows that holistic care for cancer patients can reduce the pain of patients. Compared with non-cancer patients, cancer patients are more prone to symptoms such as nausea, vomiting and constipation. This may be caused by the long-term negative emotions of cancer patients. At present, the research on the factors related to cancer pain shows that depression is related to chemotherapy reaction, side effects of chemotherapy, anti-tumor drugs and long treatment time (14, 15). In addition, the study found that cancer pain was related to many factors, such as environment, social pressure and subjective consciousness of patients. Therefore, nursing intervention for cancer pain can effectively improve the quality of life of patients with advanced cancer.

In this paper, medical image fusion technology was used to screen patients with gynecological malignant tumors in a third-class A hospital and explore the application of ERAS concept in clinical nursing of advanced cancer pain. A total of 80 patients were divided into two groups, including 40 in the experimental group (M group) and 40 in the control group (N group). Among them, group M adopted ERAS concept for pain management, that is, through prevention, timely and multi-mode analgesia. In group N, pain management was carried out by traditional methods, that is, analgesia on demand. Nine patients were selected from each group for data analysis.

The content of pain care in ERAS concept mainly includes: (1) improve the level of mental health: it can reduce patients’ fear of cancer drug treatment and related nursing measures through psychological intervention and other methods, so that they can actively cooperate with treatment. (2) Relieve bad emotions: it can strengthen communication with patients and their families, so that they can understand that cancer pain is not a physiological phenomenon but a pathological phenomenon. (3) Alleviate adverse reactions: it can give active and effective intervention measures to cancer pain. (4) It can provide early intervention for cancer patients. (5) Provide relevant health education: it can encourage patients to participate in health education activities to enhance their awareness of self-care.

Inclusion criteria: female patients over 18 years old; All patients were diagnosed as malignant tumors; Patients who have received one course of treatment or longer need further treatment; Have clear perception and the ability to answer questions; Take the initiative to be investigated.

Exclusion conditions: unable to cooperate with the investigation; Patients with nerve and digestive tract tumors.

Observation method: The NRS (numerical rating scale) score, KPS score, pain effect and adverse reactions of the patients were recorded truthfully, and the curative effects were compared between the groups.

The pain score was evaluated by NRS evaluation method, and the pain of patients was evaluated by professional medical staff according to NRS standards. Among them, 0 indicates no pain, 1-3 indicates mild pain, 4-6 indicates moderate pain, and 7-10 indicates severe pain.

Pain impact score: Pain impact includes 7 pain impact shadows of patients’ ability of daily living (A), life enjoyment (B), mood (C), sleep quality (D), walking ability (E), working ability (F), and relationship with others (G). The pain factor scores and total scores are recorded during the test, and the differences between the groups are compared. The score of each item is 0-10, the lowest score is 0, the best score is 10, and the total score of all items is 70.

KPS (Karnofsky) score is a comprehensive scoring standard for the physical condition of tumor patients. The total score is 100 points, divided into 10 grades, with 0 point for death and 10 points for dying. 20 points indicates that the patient is seriously ill and needs active hospitalization. A score of 30 indicates that life is extremely hard to take care of. A score of 40 indicates that they cannot take care of themselves and need special care, a score of 50 indicates that they often need care, and a score of 60 indicates that they sometimes need help, but most of the time they can take care of themselves. 70 points refer to self-sufficiency but unable to continue normal life or work. 80 points refers to having certain symptoms or signs, barely able to exercise normally, and 90 points refers to being able to exercise normally, with mild symptoms and signs. A score of 100 indicates that everything is normal without any signs or symptoms. According to the scores of patients’ actual conditions, the differences between the two groups were compared.

### 3.2 Comparison of scores of various factors before treatment

**Table 1** shows the score comparison of various factors before treatment. Before treatment, the NRS score of group M was 7.11, and that of group N was 6.93, P=0.793>0.05, with no statistical difference. Before treatment, the pain impact score of group M was 39.07, and the score of group N was 38.92, P=0.836>0.05, with no statistical difference. Before treatment, the KPS score of group M was 67.92, and the score of group N was 67.13, P=0.509>0.05, with no statistical difference. It showed that there was no significant difference in various factors between M group and N group before treatment.

**Table 1:** Comparison of scores of various factors before treatment.

	Group M	Group N	P
NRS score	7.11	6.93	0.793
Pain impact score	39.07	38.92	0.836
KPS score	67.92	67.13	0.509

### 3.3 Comparison of NRS scores after treatment

**Figure 3:**
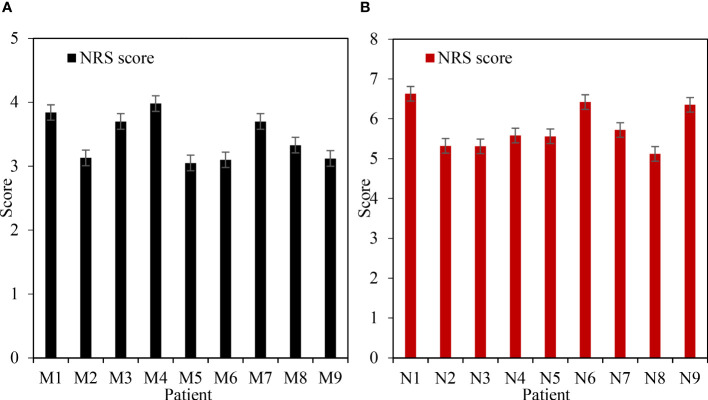
Comparison of NRS scores after treatment. **(A)** NRS score of group M after treatment. **(B)** NRS score of group N after treatment.

**Figure 3** shows the comparison of NRS scores after treatment. **Figure 3A** shows Group M and **Figure 3B** shows Group N. **Table 2** shows the NRS score t-test. Under the treatment of ERAS concept, the NRS score of patients was between 3 and 4, with an average score of 3.44. Under the treatment of traditional methods, the NRS score of patients is between 5 and 7, with an average score of 5.78. Through t-test analysis, there was a significant difference in NRS scores between group M and group N after treatment (P=0.012<0.05).

**Table 2:** NRS score t test.

	Group M	Group N
Average value	3.44	5.78
Standard deviation	0.344	0.518
P value	0.012

### 3.4 Comparison of pain impact scores after treatment

**Figure 4:**
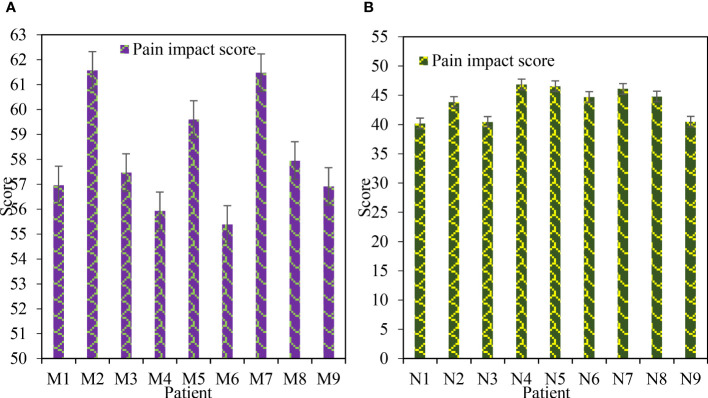
Comparison of pain impact scores after treatment. **(A)** Postoperative pain impact score in group M. **(B)** N group postoperative pain impact score.

**Figure 4** shows the comparison of pain impact scores after treatment. **Figure 4A** shows Group M and **Figure 4B** shows Group N. **Table 3** shows the t-test of pain impact score. Under the treatment of ERAS concept, the pain impact score of patients ranged from 55 to 62, with an average score of 58.14. Under the treatment of traditional methods, the pain impact score of patients ranged from 40 to 47, with an average score of 43.79. Through t-test analysis, there was a significant difference in pain impact score between group M and group N after treatment (P=0.023<0.05). It showed that the improvement of pain score in group M was better than that in group N.

**Table 3:** Pain impact score t-test.

	Group M	Group N
Average value	58.14	43.79
Standard deviation	2.13	2.57
P value	0.023

### 3.5 Comparison of pain influencing factors after treatment

**Figure 5:**
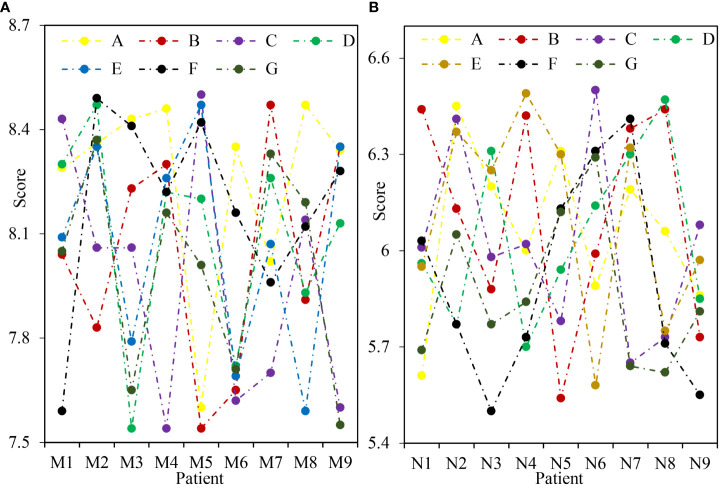
Comparison of pain influencing factors after treatment. **(A)** Factors influencing postoperative pain in group M. **(B)** Factors influencing postoperative pain in group N. A refers to the patient's daily living ability, B refers to the patient's enjoyment of life, C refers to the patient's mood, D refers to the patient's sleep quality, E refers to the patient's walking ability, F refers to the patient's working ability, and G refers to the relationship between the patient and others.

**Figure 5** shows the comparison of pain influencing factors after treatment. **Figure 5A** shows Group M and **Figure 5B** shows Group N. **Table 4** shows the mean score t-test of pain influencing factors after treatment. It can be seen from the figure that the scores of pain influencing factors in group M treated with ERAS concept are between 7.5 and 8.5. The scores of pain influencing factors in group N with traditional methods were between 5.5 and 6.5. It can be seen from the table that under the treatment of ERAS concept, the daily living ability score is 8.26 and the life enjoyment score is 8.04. Emotional score was 7.96, sleep quality score was 8.09, and walking ability score was 8.07. The score of work ability is 8.18, and the score of relationship with others is 8.00. Under the treatment of traditional methods, the daily living ability score was 6.06, the life enjoyment score was 6.11, and the emotional score was 6.02. The sleep quality score was 6.05, and the walking ability score was 6.11. The score of work ability is 5.90, and the score of relationship with others is 5.87. The factors affecting pain in group M and N were analyzed by t test, and there was significant difference (P<0.05).

**Table 4:** Average score t-test of pain influencing factors after treatment.

	Group M	Group N	P value
A	8.26	6.06	0.042
B	8.04	6.11	0.037
C	7.96	6.02	0.045
D	8.09	6.05	0.031
E	8.07	6.11	0.043
F	8.18	5.90	0.029
G	8.00	5.87	0.025

### 3.6 Comparison of KPS scores after treatment

**Figure 6:**
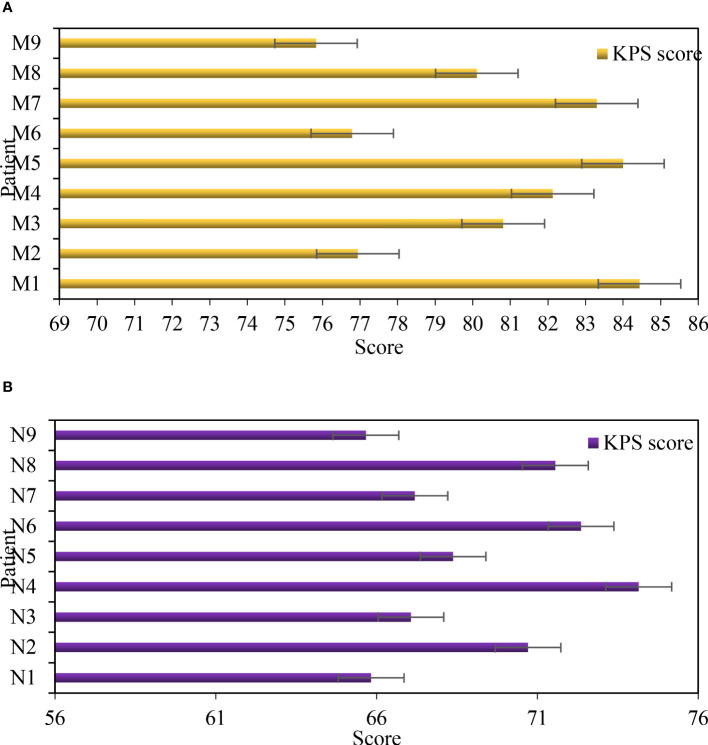
Comparison of KPS scores after treatment. **(A)** KPS score of group M after treatment. **(B)** KPS score of group N after treatment.

**Figure 6** shows the comparison of KPS scores after treatment. **Figure 6A** shows Group M and **Figure 6B** shows Group N. **Table 5** shows the KPS score t-test. Under the treatment of ERAS concept, the KPS score of patients was between 75 and 85, with an average score of 80.48. Under the treatment of traditional methods, the KPS score of patients is between 65 and 75, with an average score of 69.21. Through t-test analysis, there was a significant difference in KPS scores between group M and group N after treatment (P=0.019<0.05).

**Table 5:** KPS score t test after treatment.

	Group M	Group N
Average value	80.48	69.21
Standard deviation	3.10	2.89
P value	0.019	

ERAS concept emphasizes the concept of symptomatic treatment according to the individual conditions of patients, and adopts appropriate and effective methods to solve the symptoms caused by various diseases, such as pain, anxiety, etc. This can achieve the purpose of alleviating pain, improving the quality of life and survival probability of patients. Different from the traditional concept, ERAS concept emphasizes the combination of multiple methods in the treatment process. Among them, analgesics are most widely used in gynecological malignant tumors, but they are characterized by large side effects, high price and poor compliance of patients. The analgesic effect of opioid drugs in ERAS concept is significantly better than that of traditional opioid analgesics and local anesthetics, and its side effects are less. Therefore, it can effectively relieve the pain symptoms of patients, improve the quality of life and survival probability of patients.

To sum up, ERAS concept can be adopted to have great advantages in clinical nursing of patients with advanced cancer pain of malignant tumors, which can well relieve the pain of patients.

## 4 Conclusions

ERAS concept refers to patient-centered, comprehensive and systematic provision of high-quality services for patients. It includes health education, psychological support, related drugs, nursing, and other aspects. At the same time, it also takes into account the participation of patients and their families in the formulation of treatment plans and the whole process of treatment, so as to reduce and eliminate the cancer pain symptoms of patients and improve the quality of life. The application of ERAS nursing intervention in patients with advanced cancer pain has positive significance. First of all, patients with advanced cancer pain need professional guidance, so as to reduce their bad emotions and alleviate their pain. Secondly, pain patients should master the correct medication methods and precautions when using analgesics, which is also an education for patients and their families. ERAS nursing intervention can also relieve patients’ pain symptoms and help patients improve their understanding of cancer pain. Humanized services are provided according to the needs of patients, which can measure blood pressure for patients and provide them with a comfortable rest environment. It is necessary to patiently explain the knowledge about pain to the patient and pass on the correct self-management method of cancer pain. It should inform patients how to prevent adverse reactions of analgesics and guide them to use analgesics reasonably. ERAS nursing intervention can help patients with advanced cancer pain to reduce and eliminate pain stimulation, improve quality of life, prolong survival time, and reduce damage to other organ functions. However, due to the limitations of time and technology, this paper has not carried out a detailed analysis of the problems encountered in the research of cancer pain, which would be further discussed in the future.

## Data availability statement

The original contributions presented in the study are included in the article/supplementary material. Further inquiries can be directed to the corresponding author.

## Ethics statement

The studies involving human participants were reviewed and approved by Ethics Committee of Chenzhou First People’s Hospital (AF/SOP-EC-N14-4.0/02). Written informed consent for participation was not required for this study in accordance with the national legislation and the institutional requirements.

## Author contributions

HL: Conceptualization; methodology; software. YL: validation; formal analysis; investigation. Both authors contributed to the article and approved the submitted version.
